# An observational study of the presence and variability of the microbiota composition of goat herd milk related to mainstream and artisanal farm management

**DOI:** 10.1371/journal.pone.0292650

**Published:** 2023-10-05

**Authors:** Rita A. H. Hoving-Bolink, Adriaan F. G. Antonis, Marinus F. W. te Pas, Dirkjan Schokker

**Affiliations:** 1 Wageningen Livestock Research, Wageningen, The Netherlands; 2 Wageningen Bioveterinary Research, Lelystad, The Netherlands; University of Illinois, UNITED STATES

## Abstract

Goat milk is produced on mainstream and artisanal farms. It was expected that the farm management may influence the microbial population of the milk. Therefore, we investigated the bacterial content and microbiota composition of raw milk in relation to Dutch goat farm management. After amplicon sequencing we analyzed the taxa at phylum and genus levels, and used the relative values enabling to provide information about the variation among the different samples. On ten farms our results indicated that the number of bacterial colony forming units and microbiota composition of the milk, directly after milking was variable among farms and not related to the farm management system. At the phylum level the phyla *Firmicutes*, *Actinobacteria*, *Proteobacteria*, and to a minor extend *Bacteriodota* were the dominant phyla in the raw goat milk, together usually comprising 90% of the total bacterial phyla. The most dominant genera were *Staphylococcus*, *Pseudomonas*, *Lactococcus*, *Microbacteria*, *Acinetobacteria*, and *Corinebacteria*. The number of bacterial phyla and genera does not differ between the mainstream and artisanal farms, although the Shannon index may be numerically higher in the mainstream farms as compared to artisanal farms. In addition, the variability is higher among artisanal farms, which may be due to less standardization of the management. The milk microbiota composition differed among farms. Repeated sampling of a farm showed that this changed over time. The lactic acid producing bacteria showed a similar pattern. Variable microbiota richness amount and diversity of microorganisms were present in different farming systems. We concluded that farm-specific management and sampling moment were the major determining factors for the milk microbiota composition.

## Introduction

Milk is an important dietary component for newborn and young mammals, including humans. Next to cow milk, goat and sheep milk are important livestock dairy products. Although it is not necessarily an alternative for people with cow milk allergy, goat milk can have specific advantages over cow milk. The protein, fatty acid, lactose, vitamins, and mineral profiles of goat milk are different from that of cow milk [1–6].

Goats in the Netherlands are kept on mainstream farms, organic farms and artisanal farms. Mainstream farms keep goats indoors and deliver their milk to dairy processing industry. On the other hand, artisanal farms often enable grazing of the goats and use the milk (raw or pasteurized) mostly for cheese production on their own location. One of the advantages of raw milk is the presence of antibodies in the milk [7]. The risk of using raw milk may be contamination with unwanted bacteria from the environment. For good microbial milk quality it is important to quickly cool the milk and keep it refrigerated. For healthy cheese production from raw milk it is important to quickly acidify the milk as the first step in the cheese making process. Starting cheese production immediately after milking with fast growth of desired lactic acid-producing bacteria prevents and suppresses the growth of spoilage and pathogenic microorganisms because of the rapid acidification [8]. Measuring the bacterial colony forming units in bulk milk samples provides information of bacteria able to grow on agar plates, which is often referred to as the total bacterial content of the milk. However, this does not provide information about the bacterial composition of the milk microbiota. This can be investigated by amplifying and sequencing the variable 16S rRNA gene composition [9, 10].

The objectives of this research were to investigate (1) the potential relation between the bacterial abundance in goat milk measured as colony forming units and different goat farm management systems in The Netherlands, i.e. mainstream and artisanal farm management systems; (2) the raw goat milk microbiota diversity in Dutch goat milk samples of the different farm management systems, and whether this goat milk microbiota was stable over time on a given farm; and (3) which lactic acid producing bacteria were present in the raw goat milk of the two farm management systems.

## Materials and methods

### Farms and milk sample collection

Goat milk was collected at ten farms. The involved farmers were transparently informed about the purpose of this research and have freely given full consent for taking and using the samples. Afterwards they received a written non-scientific report and online meeting to discuss the results. Four farms were identified as mainstream farms and six farms as artisanal farms (Table 1). The selection criteria for the farms as either mainstream or artisanal farms were that they represent good examples of Dutch mainstream or artisanal milk goat farms. More specifically, the artisanal farms were selected on the use of Dutch rare breeds using pedigree breeding. Milking the goats was mandatory for selection. Both criteria also met with the mainstream farms. Artisanal farms used the raw milk to prepare on-farm goat cheese. All mainstream farms sold the raw milk to a milk factory. All farmers participated voluntarily in the investigation. In 2020 a total of 569 milk goat farms were recorded in The Netherlands (Dutch office for statistics (https://www.cbs.nl/), part of the Dutch Ministry of Economic Affairs and Climate). The number of goats on a farms was growing: from 2000 to 2020 from 313 to 1177 goats per farm. Over all milk goat farms had on average 837 animals. Of these milk goat farms 71 farm were noted as organic. Mainstream and artisanal farms differ in farm management, number of goats at the farm, and other farm-specific characteristics (Table 1; S1 File). In general, mainstream farms keep the goats inside barns while most artisanal farms allow grazing of the animals. Therefore, sun exposure, environmental temperature, humidity, nutrition (fresh gras or not, and the amount of concentrate) differ between the two farm management systems. Due to size differences of mainstream and artisanal farms the milking equipment usually differs. Exceptional, farm 8 applied hand milking. Finally, while the mainstream goat farms have milk hygiene protocols, artisanal farms had not. Farms 3 and 10 used more than one specific goat breed. More details can be found in S1 File.

**Table 1:** Characteristics of the farms.

Farms	Farm type^1^	Herd size	Breed	No. in milk sample^2^; (number of samples)	Milk tank^3^	Sampling months
**Farm 1**	A	75	Dutch Toggenburger	15 (8)	No	June (2x), August, October
**Farm 2**	A	100	Dutch Pied Original Goat	15 (4)	Yes	June, October
**Farm 3**	M	210	Dutch Nubian / Dutch Pied Original Goat / Dutch White Goat / Dutch Toggenburger	32 (5)	Yes	August, September
**Farm 4**	A	145	Dutch White goat /	32 (3)	Yes	August
**Farm 5**	M	500	Dutch Dairy Goat	60 (3)	Yes	August
**Farm 6**	A	7	Dutch Toggenburger	4 (2)	No	August
**Farm 7**	M	1195	Dutch Dairy Goat	80 (3)	No	September
**Farm 8** ^**4**^	A	7	Dutch Toggenburger	7 (2)	No	October
**Farm 9**	A	12	Dutch White Goat	3 (2)	No	October
**Farm 10**	M	700	Dutch Dairy Goat / Alpine	48 (3)	Yes	October

1: A: artisanal; M: mainstream

2: Number of goats contributing to the milk sample (i.e. number of goats being milked at the time of sampling).

3: Milk was collected from a collection device into a cooling tank with one or more milking’s accumulated

4: Farm 8 was the only farm where goat were milked by hand

During the morning round of milking milk samples were collected directly after milking from the milk collection device. All samples were collected and analyzed in duplicate. Gloves were used with sample collection to prevent contamination with human DNA. A warm milk sample was collected from the milk collection device. Two samples from each farm were used for farm comparisons. If a milk tank is available a sample is collected from the tank. Six farms had a cooled milk storage tank with mixed milk from one to three previous milking turns. A milk tank was also sampled at the same day as the sampling of the milk directly after milking. Farms 1, 2, and 3 were repeatedly sampled, respectively four (farm 1) and two times (farms 2 and 3). Samples used for microbiota analysis were collected in a 10 ml Falcon tube, immediately frozen on dry ice, and stored at -80°C until analysis (Wageningen Bioveterinary Research (WBVR), Lelystad, The Netherlands). Samples used for bacterial colony forming units analysis were placed in 100 ml flasks, and kept cooled until colony forming units (CFU) analysis (QLIP, Zutphen, The Netherlands, https://www.qlip.com/nl/).

### Raw goat milk colony forming units and microbiota analysis

The milk colony forming units (CFU) was analyzed by agar plate count (QLIP, Zutphen, The Netherlands). Bacterial colony forming units were taken as a quantification of the total number of bacteria in the milk. However, it should be noted that in the CFU only aerobic bacteria that are able to grow on agar will be cultured. The milk colony forming units were measured by QLIP using the total colony count method for aerobe culturable bacteria (https://www.qlip.com/nl/). The method is specific for culturing the bacteria of milk or milk products, cheese and cheese products. The plate count method was performed conforming to the NEN-EN-ISO 4833–1 regulations. Petri dishes with plate count milk agar were mixed with a 10 times dilution series of the samples and cultured at 30°C for 72 hours. The measurements were done in duplicate.

The milk microbiota composition of 35 milk samples were analyzed. Bacterial DNA was extracted from the goat milk microbiota with the PureLink Genomic DNA Kit (Invitrogen, ThermoFisher, Waltham, Massachusetts; https://www.thermofisher.com/document-connect/document-connect.html?url=https://assets.thermofisher.com/TFS-Assets%2FLSG%2Fmanuals%2Fpurelink_genomic_man.pdf) using 200 μl milk. For elution the kit elution buffer was replaced with 100 μl EB buffer of Qiagen (Venlo, The Netherlands, #19086).

Samples were amplified and sequenced by targeted-amplicon 16S sequencing on the MiSeq, where the forward primer CVI_V3-F (5′CCTACGGGAGGCAGCAG-3) and reverse primer CVI-V4_R (5′-GGACTACHVGGGTWTCT-3′) were used. The amplicons conditions used were described previously [11], briefly, 98° C for 2 m, followed by 20 cycles of 98° C for 10 s, 55° C for 30 s, 72° C for 10 s, and finally by 72° C for 7 min. The triplicate PCR products were pooled, checked on TapeStation (Agilent, United States), and after barcoding per sample, subsequently sequenced on a MiSeq sequencer (Illumina Inc., San Diego, CA) using a V3 paired-end 300 bp kit. Processing of sequence data was performed in R 4.0.5 [12], were amplicon sequences were demultiplexed per sample, and thereafter filtered, trimmed, error-corrected, dereplicated, chimera-checked, and merged using the DADA2 package (v.1.18.0) [13]. By using the standard parameters except for TruncLength = (180,175), trimLeft = (0), and minOverlap = 10, reads were classified against the SILVA v.138.1 database [14]. Downstream analyses were performed on taxa levels, phylum and genus, and used relative abundance values. The diversity as described by the Shannon index [15] was calculated by using the vegan package (v2.5–7) [16] within the R environment. The Shannon diversity index (H) is commonly used to characterize species diversity in a community. The Shannon index accounts for both abundance and evenness of the microbiota species present. The top ten bacterial phyla and genera are presented. The bacterial data were analyzed with a Wilcoxon Rank Sum and Signed Rank Test. The variability was measured with multivariate homogeneity of groups dispersions. Sequencing data generated in this study have been deposited in the NCBI Sequence Read Archive database under BioProject Accession Number PRJNA948035.

## Results

### Colony forming units

**Fig 1:**
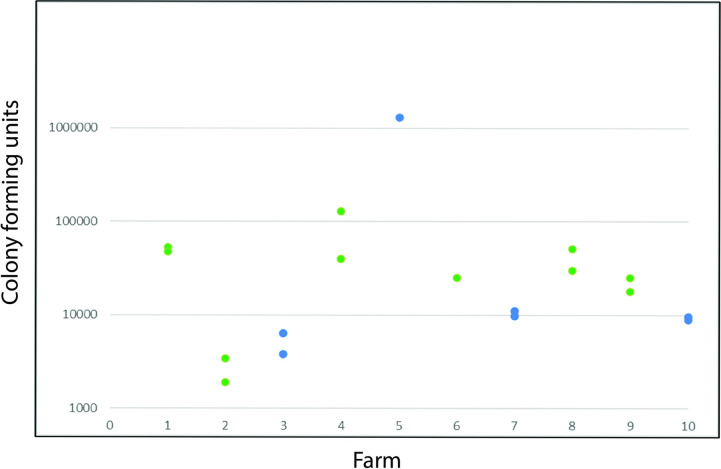
Goat milk colony forming units of the ten farms. The selected samples are the same as in Figs 2 and 3. Each farm is represented with two independent samples. All raw data can be found in Table S2 in S2 File. Blue dots represent mainstream farms, green dots represent artisanal farms. Please note two missing values of farms 5 and 6, respectively, due to unreliable dilution series.

**Fig 2:**
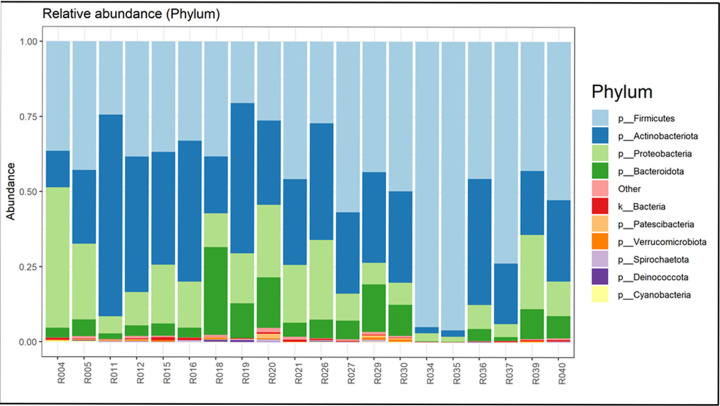
Relative abundance of the goat milk microbiome bacteria at the phylum level of each farm. Each farm is represented by a duplicate sample. The top ten phyla are indicated; all other phyla are represented by the term “other”. Farm 1: R029, R030; Farm 2: R004, R005; Farm 3: R015, R016; Farm 4: R011, R012; Farm 5: R018, R019; Farm 6: R020, R021; Farm 7: R026, R027; Farm 8: R034, R035; Farm 9: R036, R037; Farm 10: R039, R040.

**Fig 3:**
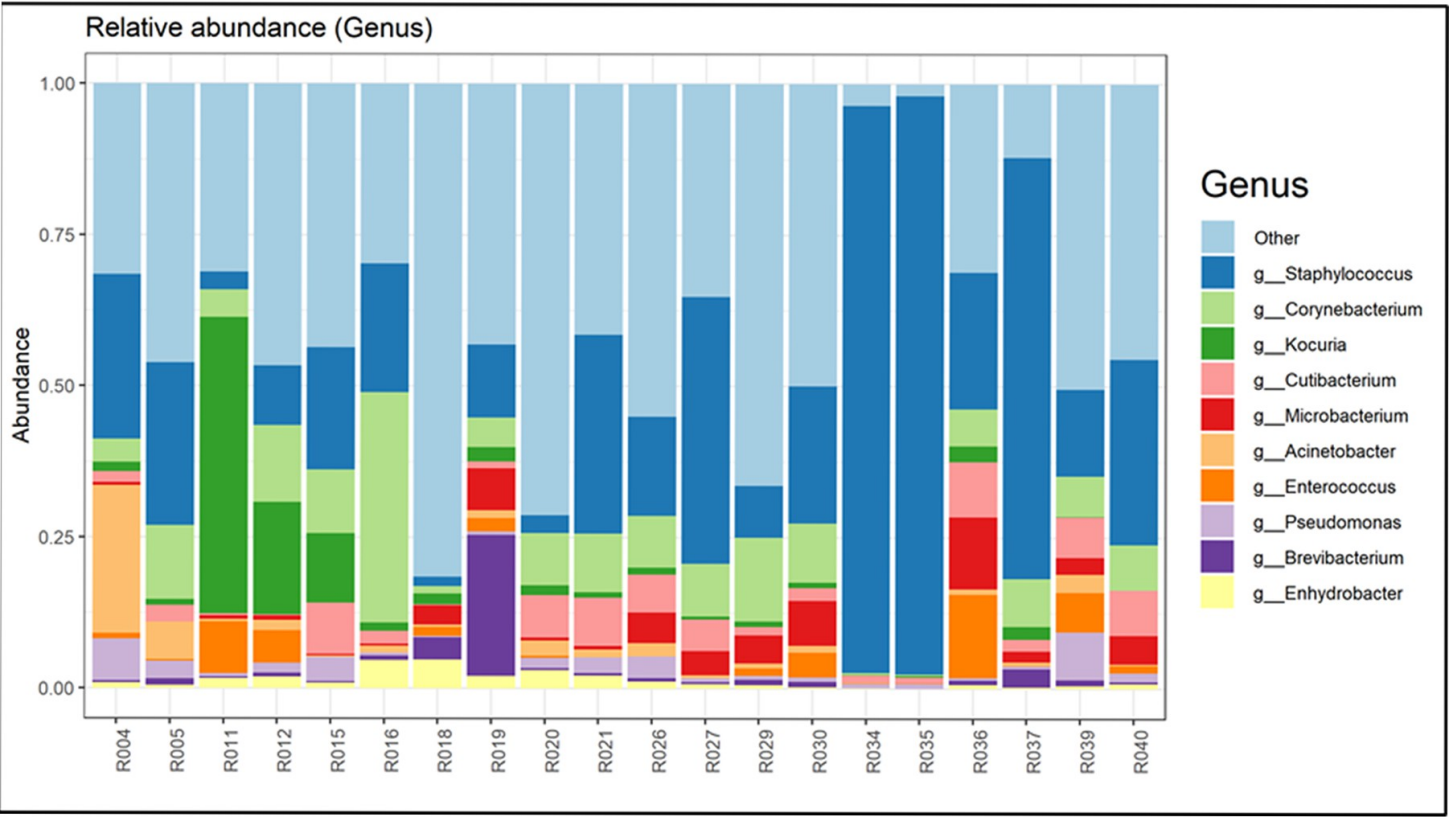
Relative abundance of the goat milk microbiome bacteria at the genus level of each farm. Each farm is represented by a duplicate sample. The top ten genera are indicated; all other genera are represented by the term “other”. Farm 1: R029, R030; Farm 2: R004, R005; Farm 3: R015, R016; Farm 4: R011, R012; Farm 5: R018, R019; Farm 6: R020, R021; Farm 7: R026, R027; Farm 8: R034, R035; Farm 9: R036, R037; Farm 10: R039, R040.

Fig 1 shows the total agar culturable aerobic bacterial content measured as CFU of goat milk samples of the ten farms. The samples in Fig 1 are the same as analyzed for the microbiota in Figs 2 and 3. Fig 1 shows that the CFU of the samples of a specific farm are closely related, while there is some variation among farms. The farms with the lowest CFU are farms 2 and 3, a mainstream and an artisanal farm. Farms 4 and 5 have a CFU sample higher than 100,000, also representing a mainstream and an artisanal farm.

The raw data of all samples can be found in Table S2 in S2 File. Here, on some days several farm 1 samples showed extremely high bacterial CFU. Again, all samples of farms 2 and 3 showed the lowest CFU, and farms 4 and 5 have relatively higher CFU. Table S3 in S2 File shows the data sorted for breed, Table S4 in S2 File shows the data sorted for the number of goats on a farm, and Table S5 in S2 File shows the data sorted for date of sampling. Due to the experimental design breed, farm size, and farm management could not be disentangled.

### The raw goat milk microbiota at ten Dutch farms

The microbiota in raw goat milk at the farm level was determined at the level of bacterial phyla and genera (Figs 2 and 3 –farm specific microbiota analyses can be found in S3 File, Figs S1-S10 in S3 File). The raw data reads per sample can be found in the Table S6 in S2 File. Prior to filtering we identified 7961 taxa, after sub-setting of samples, pruning, and rarefying 6738 taxa were left. The data were rarefied to 114046. The results showed both general aspects and farm microbiota fingerprints. At the phylum level the phyla *Firmicutes*, *Actinobacteria*, *Proteobacteria*, and to a minor extend *Bacteriodota* were the dominant phyla in the raw goat milk, together usually comprising over 90% of the total bacterial phyla found. The most dominant genera were *Staphylococcus*, *Pseudomonas*, *Lactococcus*, *Microbacteria*, *Acinetobacteria*, and *Corinebacteria*. The group “other”, comprising a large list of bacterial genera each with a small occurrence, showed the highest content: in many samples up to 65% of the total bacterial content (Tables S7 and S8 in S2 File).

Several bacterial groups were specific for farms. The *Actinobacteriota* showed lower abundance in the samples from farm 2 and 8, but was found up to 70% at farm 4. Please note that only the top ten bacterial groups were specified, with all other bacterial groups placed in the group indicated as other. While *Proteobacteria* were absent or at a low abundance at farm 8, at other farms the content was up to 30%. Farms 8 and 10 showed high content of *Staphylococcus*, of up to 95%.

**Fig 4:**
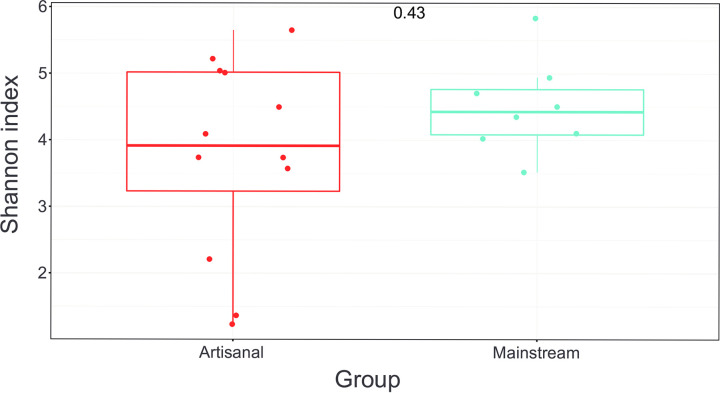
The Shannon diversity index. The box plot shows the variation among the farms in the bacterial composition of the goat milk. On average the number of bacterial genera is a little higher in the mainstream farms, while the variation is higher in the artisanal farms, but the management systems were not different. The Shannon diversity index varies from 1 to 6, where 1 means low and 6 means high balanced bacterial diversity.

**Fig 5:**
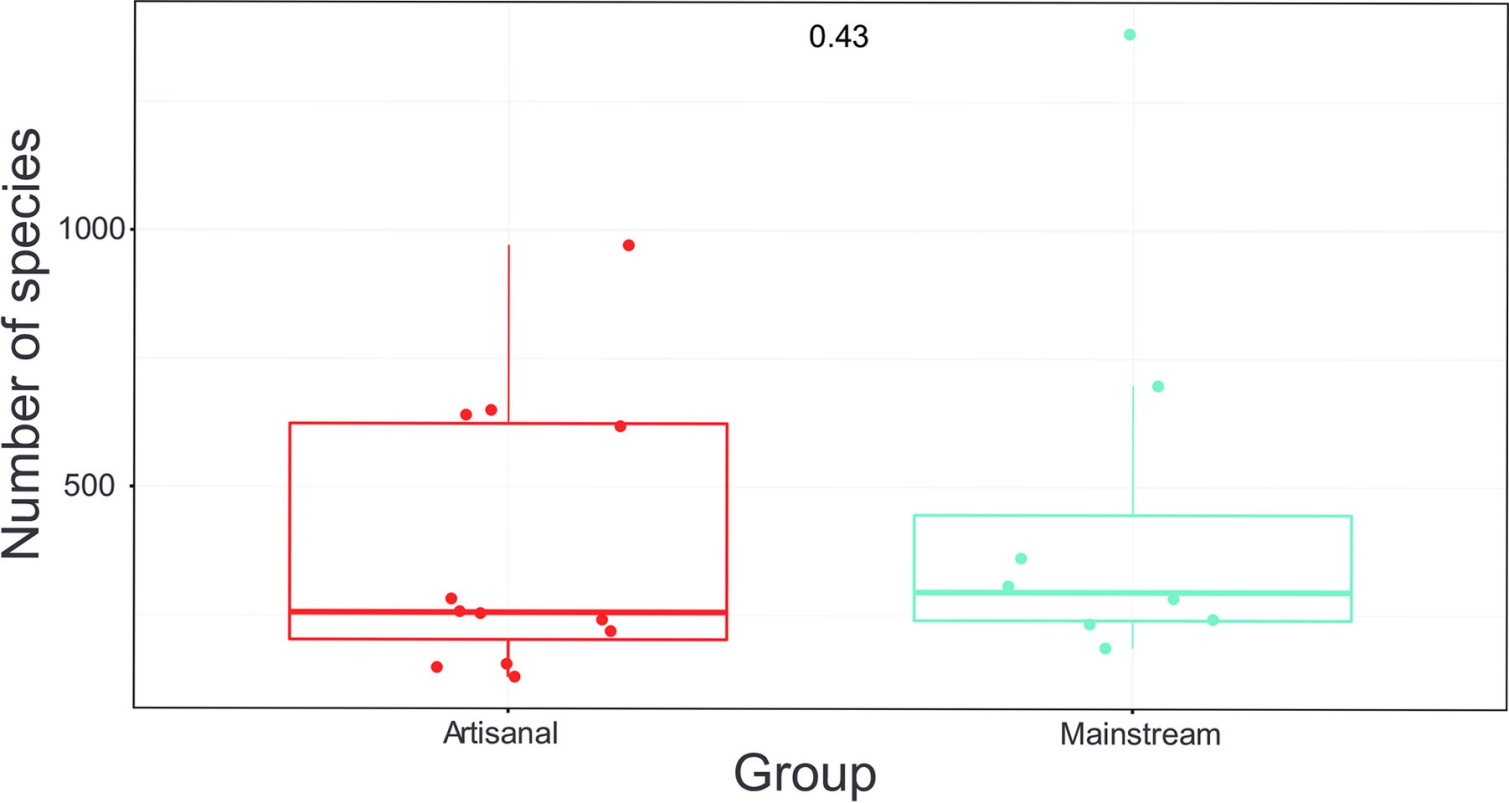
Box plot of the alpha-diversity; Observed species richness showing the variation in the total number of observed bacterial species (genera in our data). The two management systems were not different, but the Fig shows that the variation is larger among artisanal farms as compared with the mainstream farms.

The number of bacterial phyla and genera does not differ between the mainstream and artisanal farms, although the Shannon index may suggest a little higher number of bacterial genera in the mainstream farms as compared to artisanal farms. The variability measured with multivariate homogeneity of groups dispersions was higher in the artisanal farms as compared to the mainstream farms (Fig 4). The alpha variability in the milk microbiota is higher at artisanal farms as compared to mainstream farms (Fig 5). The Principal Coordinates Analysis (PCoA, multidimensional scaling) shows the similarities and dissimilarities of the goat milk microbiota among the farms (Fig S11 in S3 File). The Fig shows that the farm types do not cluster together, and the variation among the artisanal farms is larger. The Fig shows that 33.6% (21.4% + 12.2%) of the variation is explained by the first two axes.

### The raw goat milk microbiota changes over time

To study the stability of the microbiota per farm, farms 1, 2, and 3 were repeatedly sampled over time (Table 1). The farm goat milk microbiota from different sampling dates showed that the farm-specific milk microbiota is changing over time (Figs S1-S3 in S3 File). The results of the milk microbiota samples clearly showed that the samples from the same day are very similar, with a clear “fingerprint” of the farm per sample moment. However, the samples repeated over time showed that the composition of the milk microbiota changed.

**Fig 6:**
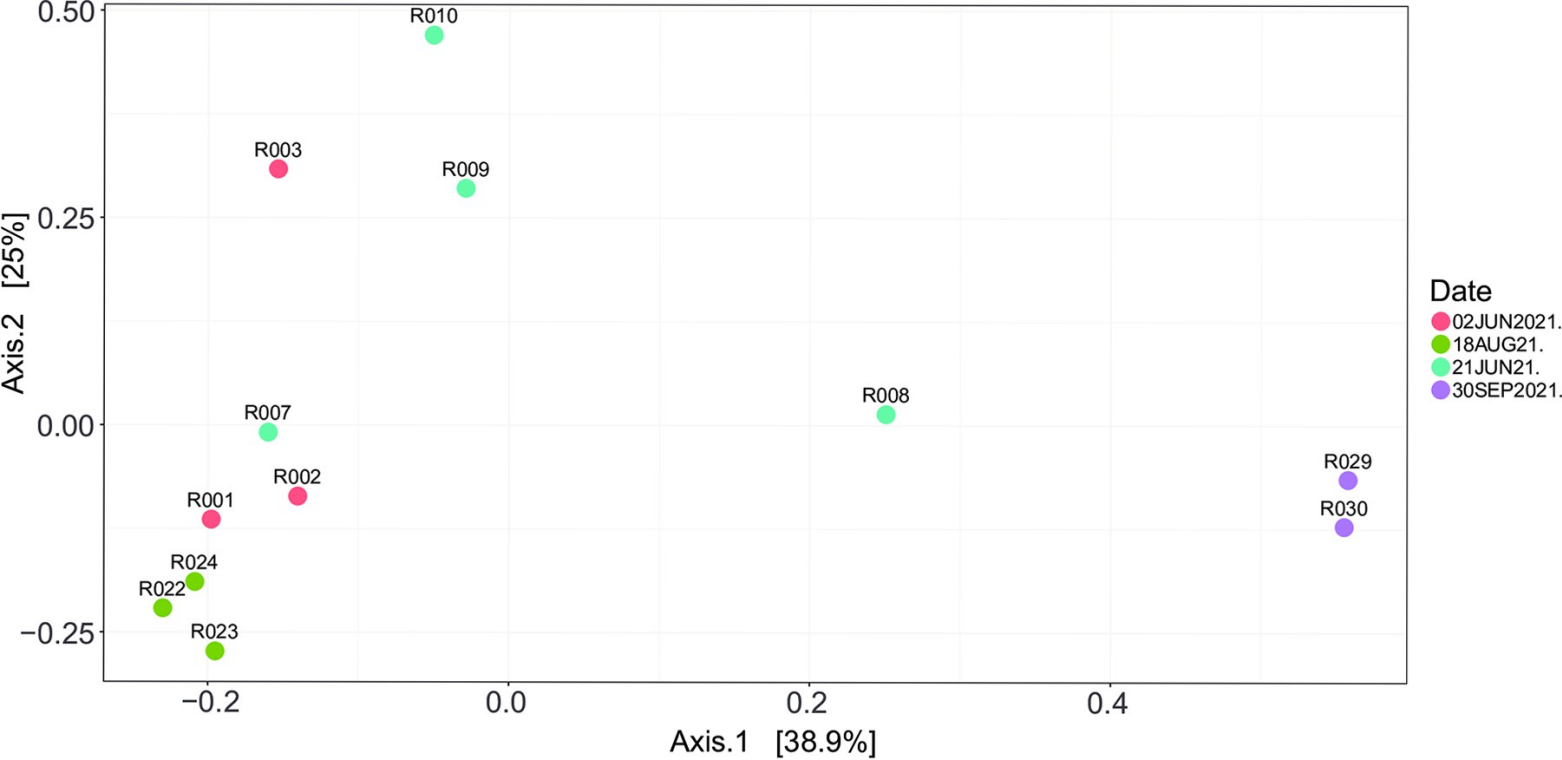
The PCoA analysis of the raw goat milk microbiota of farm 1, which has been sampled at four different days.

Farm 1 has been investigated four times. Fig 6 shows the PCoA analysis. The Fig shows that 63.9% (38.9% + 25.0%) of the variation is explained by the first two axes. The Fig shows that while samples from one sampling moment showed similar milk microbiota composition, different sampling moments showed different results. On the lower right there are two samples taken at one sampling date, which show high similarity with samples of other farms, whereas the other samples are on the lower left. The samples on the top left were warm fresh milk samples, directly after milking added to the milking device and then sampled, whereas the other samples are on the lower left.

Table S8 in S2 File shows the results per farms individually. Samples from farms at different sampling moments suggest that bacterial groups may have inverse relationships: if one group increases in content, the other group decreases, relative to the total microbiota population. Farm 1 showed remarkable variability of the *Firmicutes* over time, varying from less than 20% to over 50%. At the genus level the “other” group was up to 65%.

### The milk tank raw goat milk microbiota

Not all small scale producers have a milk storage tank. Milk tank samples showed previous milking turns at the farm of the day(s) before. In general the goat milk microbiota was highly similar with few exceptions: At farms 2, 4, 5, and 10 the milk tank samples showed increased relative content of *Firmicutes* and *Proteobacteria* as compared with the samples taken immediately after milking, while the milk tank sample at farm 10 showed the opposite changes. Farm 3 milk tank samples were similar to samples taken immediately after milking (Table S7 in S2 File; Figs S2-S5, S7 and S10 in S3 File, respectively).

### Lactic acid bacterial content of the raw goat milk

**Fig 7:**
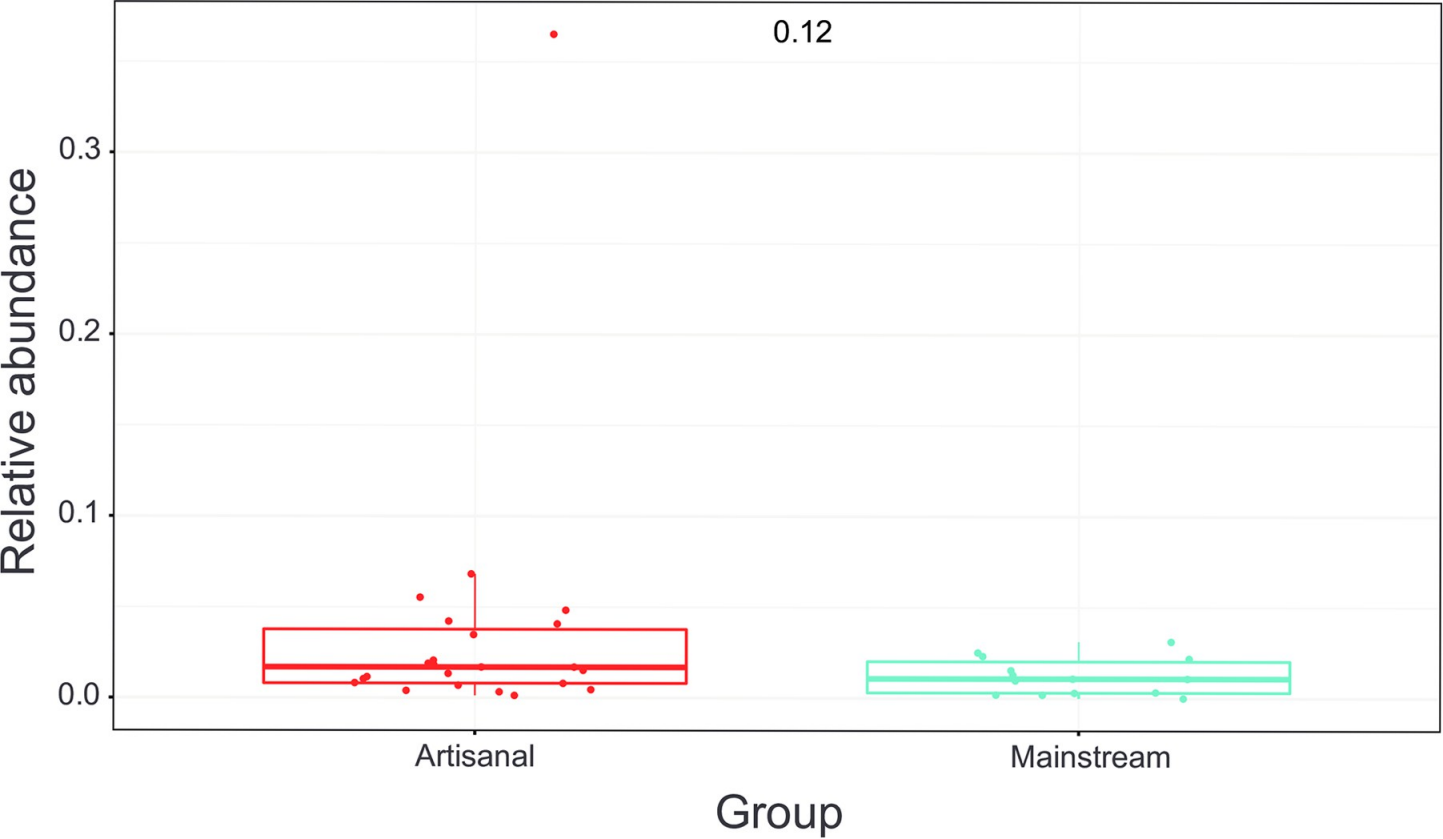
Box plot showing the observed variation of the lactic acid synthesizing bacteria in the goat milk microbiome. The average value of the two management systems is similar, but the variation is higher in the artisanal farms, varying from very high (up to above 6% of the total bacterial content) to very low (almost zero).

Adding a starter to the milk at the start of the cheese making process affects the bacterial population. Lactic acid bacteria (LAB) acidify the raw milk as a first step and the question was if some farms could have already LABs in the fresh milk. The abundance of LAB’s were highly variable among the farms. However, mainstream and artisanal farms did not differ (Fig 7). Similar to the microbiota composition, the variability of the LAB was higher in artisanal farms as compared with mainstream farms. We found artisanal farms with high content of LAB, even above 6% of total bacterial content. However, we also found artisanal farms with very low content of LAB, down to almost 0%.

## Discussion

This exploratory research investigated the amount and diversity in the relative abundance of bacteria (top eight at phylum level and top eleven at genus level) in raw goat milk per farm and the variation between farms. We used the 16S rRNA gene fragment amplification and sequencing method as a method used in almost all similar research reports. Amplicon amplifying and sequencing, like 16S rRNA, mainly answers the question; “who is there”? However, this method has its limitations since bacterial taxa, such as staphylococcus associated sequences, may be under represented. Information of different microbes, often the majority will be of bacterial origin, will surface. However, this will not aid in the understanding of the functioning of the microbiota composition. This requires the investigation of the expressions of the bacterial genomes such as metatranscriptomics, metaproteomics, and metametabolomics. While metagenomics provides more insight in the capability of the microbiota: “what can they do?”, metatranscriptomics and metaproteomics shows the expression of genes of the whole microbiota community: “what do they do”, and “how do they respond”? In our study we only scratch the surface in generating answers of which microbes are present in goat milk in Dutch farms that fits the nature of our exploratory research.

We focused on the most abundant bacteria because the total list of bacteria contains many minor phyla and genera. Specifically, the amount of lactic acid producing bacteria in this natural flora was investigated. This provides insight in the variation in microbiota at farms. It should be noted that samples were farm samples and not individual animal samples. Therefore, individual animal variation cannot be investigated.

### Farm characteristics and their interactions

Among the ten farms of the study there were six artisanal farms and four mainstream farms. Among the ten farms major environmental differences for the goats may add to the explanation of the differences in the milk microbiota. For example, most mainstream farms keep the goats inside barns while most artisanal farms allow grazing of the animals. Thus, sun exposure, environmental temperature, humidity, nutrition (fresh grass or not) are among the environmental differences that could explain part of the goat milk microbiota differences. It should be noted that sometimes the artisanal goats also were located in the barn. So, these differences could not solely explain the observed differences. Another farm specific difference include the method used for milking. Apart from using different equipment related to the number of animals milked on the farm, one farm (farm 8) applied hand milking. Although speculative, Staphylococcus bacteria potentially from human or goat skin, were observed in the milk microbiota. Finally, Among the herds enrolled in this study, while four mainstream goat farms had milk hygiene protocols, the six artisanal farms did not have such protocols.

In these Dutch farms five goat breeds were used. The Dutch Dairy Goat breed was used on most mainstream farms, although one mainstream farm uses rare Dutch breeds. The artisanal farms were selected on the use of Dutch rare breeds. There were two farms with multiple breeds: one mainstream farm used both Dairy Milk Goat and White breeds, and farm 3 used four breeds. The mainstream farms were larger in numbers of animals than the artisanal farms (Table 1) and the management of the animals was different. This cannot be disentangled.

### Study design

The study design showed several limitations, and future studies need to have an improved study design. One limitation is the relative low number of farms in each category. Especially the variation in farm management practices among the artisanal farms makes it difficult to explain the variability among the farms for the goat milk microbiota. Another limitation are the selection criteria of the farms: farms needed to be good examples of Dutch mainstream or artisanal goat milk farms, and the artisanal farms needed to use rare breeds with pedigree breeding. These selection criteria prevents extrapolation of the results to the wider Dutch goat milk farms community that not fulfil these criteria.

Furthermore, a limitation of this study was the lack of a farmer interview about farm management practices including a hygiene protocol. Such a survey could have been used in the selection procedure of the farms, and therefore could have affected the experimental design. We recommend that future studies will start with such an interview before selection of the farms.

The number of farms is low. Especially in the light of the variation in farm management as mentioned above, the low number of farms could make statistical evaluation weak or impossible.

### Bacterial colony forming units of raw goat milk

Bacterial colony forming units are bacterial counts of culturable aerobic bacterial species. Therefore, these results may differ from the amplification and sequencing of the 16S rRNA microbiota results. All samples were below 100,000 CFU with a few exceptions. When strict milking hygiene is applied in clinically healthy goats, the total bacterial counts in milk after milking should be low. Different guidelines were presented for bacterial count threshold related to consumer health [17]. It has been shown that farm management affect the CFU [18]. Furthermore, both somatic cells counts and total bacterial counts in raw goat milk changes during lactation [19, 20].

The average bacterial colony forming units in goat milk is known to be higher than in cow milk [19, 20]. We observed high variability from 1,900 bacterial colony forming units up to 2.6 million per ml milk. High values were found in the milk of both mainstream and artisanal farms. Especially at farm 1 we observed high values (Table S1 in S2 File, samples R022 and R023). The problem of this farm may relate to the cleaning of the milking device in which the raw milk was collected. Using samples after the cleaning eliminated the difference between farm 1 and the other farms. The observed difference the breed and sampling dates factors may directly relate to this. After cleaning the milking device the Toggenburger breed used by farm 1 showed similar results to other farms that also used the Toggenburger breed. Sampling date may also indicate a difference among farms related to changes over time (see below). Thus, our empirical approach uncovered a high variability in bacterial colony forming units between and within farms, however, we could not identify the factors explaining this variation.

### Bacterial composition of the raw goat milk microbiota

The raw goat milk microbiota composition consists of a variety of bacteria with different functionalities. Bacteria such as *Lactococcus*, *Lactobacillus*, *Streptococcus*, and *Propionibacterium* may be important for fermentation of the milk [21, 22]. Bacteria like *Pseudomonas* may play a role in milk spoilage [23]. Pathogenic bacteria in the milk may include *Listeria*, *Escherichia coli*, or *Campylobacter* [24]. The latter has been found on 33% of goat farms, and *Listeria* on 9% of goat farms in a search among Dutch goat farms [25]. These bacterial genera that contain pathogenic species were found with lower abundance in our milk samples, i.e. not in the top ten. For example, only for two samples, in different farms, the Brucella genus was not zero. This concerned an artisanal farm where the average relative contribution (ARC) was 0.01% and a mainstream farm with 0.09%, averaged per group this resulted in artisanal 0.001% ARC and mainstream 0.01% ARC. Conclusions based on such low data are highly speculative. It should be noted that the 16S gene count data from the amplified and sequenced data at the genus level is not sufficient to assay pathogen-specific bacteria at the species level for food-safety risk analysis–which also requires extensive bioinformatics analysis, and metatranscriptomics, metaproteomics, and metametabolomics for investigating the microbiota functioning. However, they may be found as minority groups under the “other” group of bacteria. Goat milk may also have health promoting bacteria like Lactobacilli and Bifidobacteria [26, 27]. *Lactococcus* genera and other LAB were found, which was expected in a milk sample. We observed higher variability of LAB in artisanal farms as compared with mainstream farms. An effect of farm management on LAB content was also observed in cow milk [28].

The raw goat milk of farm 8 showed a high content of *Staphylococcus* bacteria. This farm was the only farm with hand milking. The *Staphylococcus* is a bacterium commonly found on human and animal skin [29, 30]. This can be the explanation for farm 8 that showed a high content of *Staphylococcus* bacteria. Therefore, we assume that this bacterial content can be explained by this. With a bacterial content of over 95% of the total raw goat milk shows the great impact of this milking method.

### On-farm raw milk cooling

Milk cooling after milking reduces bacterial growth. The growth of all bacterial species is slowed down, but bacterial species may differ for the rate of slowing down [31]. For most bacterial phyla and genera abundances there were no major changes, with few exceptions, such as *Pseudomonas*. This may relate to the time the milk samples were cooled in the milk tank, since the milk tank samples were taken within a few (one or two) days after milking. Especially *Pseudomonas* species responsible for milk spoilage are known to be able to grow under low temperature conditions [32]. Therefore, it seems logical that they are present at relative higher abundance in cooled milk samples.

### Farm management and the milk microbiota

There was more variation in farm management comparing artisanal farms than in mainstream farm management. S1 File shows that there is variation specifically in artisanal farms of grazing, the type of milking device, and the processing of raw milk. However, the number of farms in this study were too small for a statistical analysis, so it is difficult to draw a hard conclusion on this point. We recommend to collect more data in a survey in a future study to investigate whether these variations are real variations in farm management [33]. Farm management can be a source of variation of the milk microbiota. The artisanal farms used the raw milk for on-site cheese production. Quick addition of starter create an environment with unfavorable living condition for unwanted bacteria, such as spoilage and pathogenic bacteria [8]. The large *Lactococcus* content is specific to start this process [34]. The high bacterial colony forming units found on farm 1 may be related to the hygiene conditions. Similarly, the major differences among farms for the abundance of the lactic acid synthesizing bacteria in the raw goat milk may be an important starter for the acidification.

The major differences in milk microbiota composition among the ten farms led to the concept of the farm-specific microbiota composition. However, this farm-specific microbiota composition is not stable. Each time a farm was repeatedly sampled the microbiota composition changes, while the milk samples during milking and compared with tank milk of earlier milking’s that week, were comparable. This suggest that environment (weather, farm management, etc.) is a driver. However, the specific factors underlying this result are unknown and can be subject of further investigation. Since artisanal farms have different goat breeds it can only be speculated that farm differences may also relate to goat breed differences. However, these goat breeds were also found on some mainstream farms. Contrary to this, Tilocca et al. [35] described a stable microbiota composition both 16S rRNA gene amplification and sequencing, and metaproteomics in the goat cheese samples according to the ripening timepoint. However, although raw milk and cheese production are highly related, they are different time points. After the initiation of the cheese making process the microbiota changes [35]. While these authors conclude that the major structural rearrangements occurred in the early steps of the cheese making process, our time point is before this time point suggesting that the initiation of the fermentation towards cheese making. Indeed, these authors discuss that the raw unprocessed milk carried a high level of microbial diversity, similar to our observations. Thus, we can conclude that our results are in line with previous findings of raw goat milk. Similar conclusions were drawn in the review paper of Quigley et al. [36]. They discuss that milk, due to its high nutritional content, can support a rich microbiota, which enter the milk from a variety of sources [36]. Among these are not only beneficial bacteria, but also spoilage and pathogenic bacteria. Fortunately, we have no indication of these bacteria in our samples, although low levels of these types of bacteria may occur.

## Conclusions

This research showed that the milk microbiome of raw goat milk was highly variable among farms and among different sampling over time. Milk sampled at two consecutive days (i.e. warm milk and cooled tank milk) showed similarities. One reason for a higher variation among artisanal farms may be related to their variance in farm management.

## Supporting information

S1 File Farm-specific information. (XLSX)

S2 File Tables S1-S5: Detailed information about farms and samples selected for Mainstream (M) / Artisanal (A), Bacterial colony forming units / ml milk, Breed, Number of goats on the farm, and sampling date, respectively. **Table S6**: Reads per sample. **Tables S7 and S8**: Goat raw milk microbiome information at phylum and genera levels per sample and farm, respectively.
(XLSX)

S3 File Figs S1-S10: Goat raw milk microbiome information of farms 1 to 10. **Fig S11**: Principal Coordinates Analysis (PCoA, multidimensional scaling) analysis showing the similarities and dissimilarities of the goat milk microbiome among the farms.
(DOCX)
