# Supplementary material for: An observational study of the presence and variability of the microbiota composition of goat herd milk related to mainstream and artisanal farm management
Source: PLoS One. 2023 Oct 5;18(10):e0292650. doi: 10.1371/journal.pone.0292650 (PMC10553829; doi:10.1371/journal.pone.0292650)
Supplement: S3 File — Fig S11: Principal Coordinates Analysis (PCoA, multidimensional scaling) analysis showing the similarities and dissimilarities of the goat milk microbiome among the farms. (DOCX) [file pone.0292650.s003.docx]

**Presence and variability of the microbiota of goat milk related to farm management**

Rita A.H. Hoving-Bolink^1^, Adriaan F.G. Antonis^2^, Marinus F.W. te Pas^1^, Dirkjan Schokker^2^

*1: Wageningen Livestock Research, Wageningen, The Netherlands; 2: Wageningen Bioveterinary Research, Wageningen, The Netherlands.*

**General information**

The goat milk microbiota is an important indicator for the variation in farm management. We determined the milk microbiome on ten goat farms, four mainstream goat farms and six artisanal farms. This supplementary information provides the details of the results of the goat milk microbiota compositions of the ten farms. For further details see the manuscript. For sample numbers details and sampling moments see Supplementary file 1.

**Milk microbiome analysis**

Details per farm for the milk microbiome composition at the phylum and the genus levels, and statistical analysis of these data. For further details see the manuscript.

**Overview of the milk microbiome at the phylum and genus levels of 10 Dutch goat farms: Detailed analysis of the milk microbiome analysis per goat farm**

Three farms were sampled multiple times. Two farms had more than one goat breed. Each farm is represented by duplicate samples. For further details see the manuscript. For sample numbers details and sampling moments see Supplementary file 1.


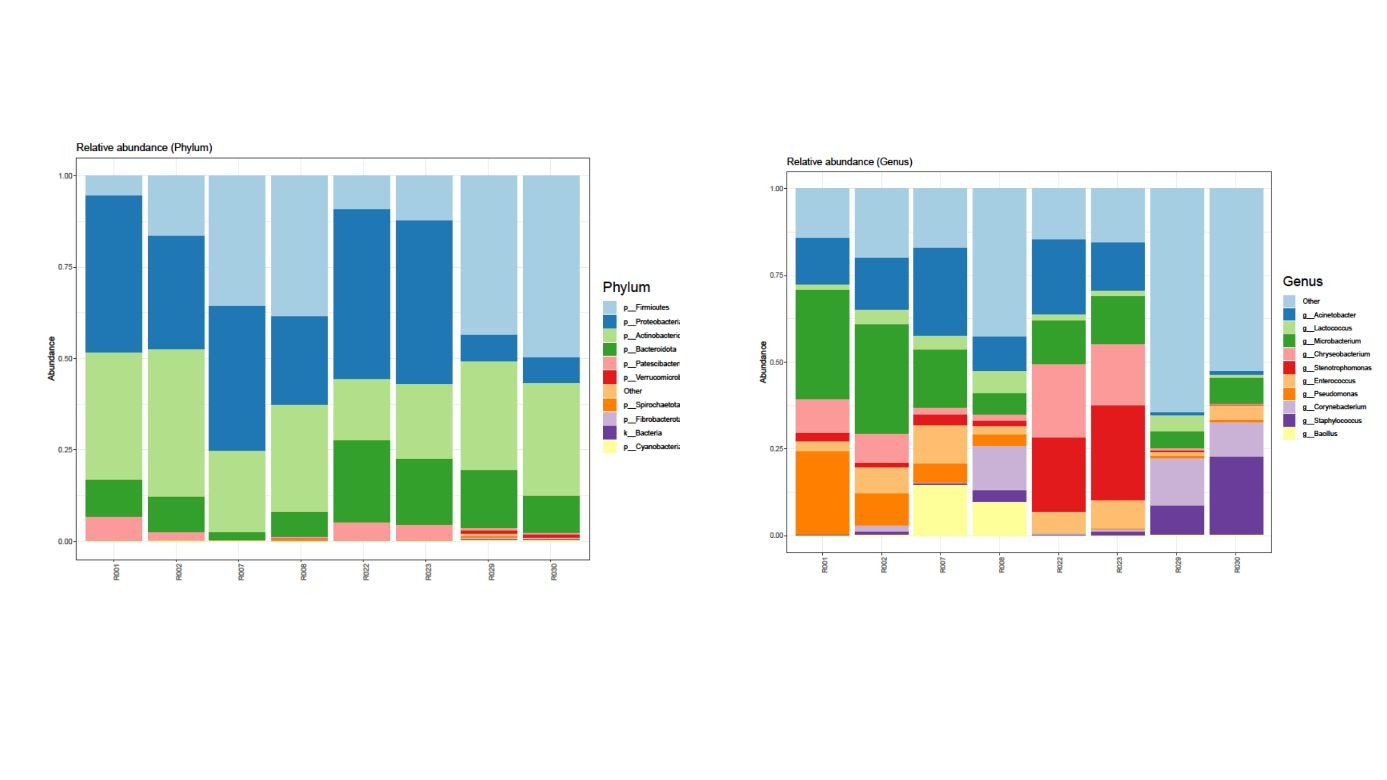


Figure S1. Data of Farm 1. Samples were taken at four sampling moments: R001 and R002 on June 2, 2021; R007 and R008 on June 21, 2021; R022 and R023 on August 19, 2021; and R029 and R030 on September 30, 2021.


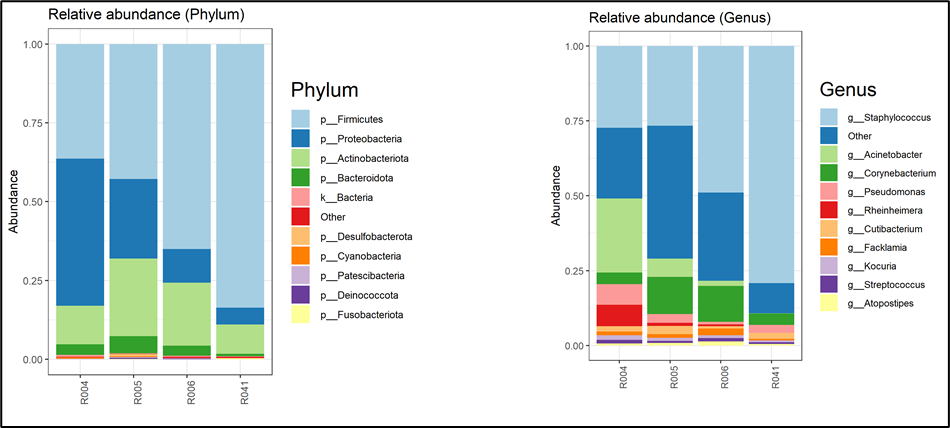


Figure S2. Data from Farm 2. Samples R004 and R005 are taken at June 20. Samples R006 and R041 were milk tank samples.


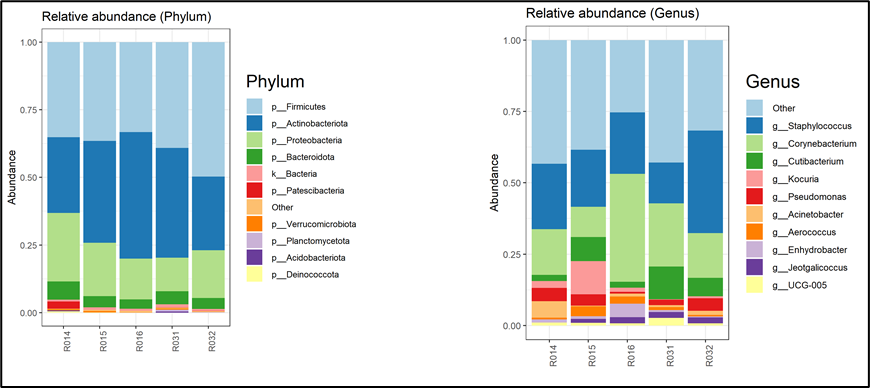


Figure S3. Data from Farm 3 samples taken at August 12 and September 30. Sample R014 is a milk tank sample containing two milking moments.


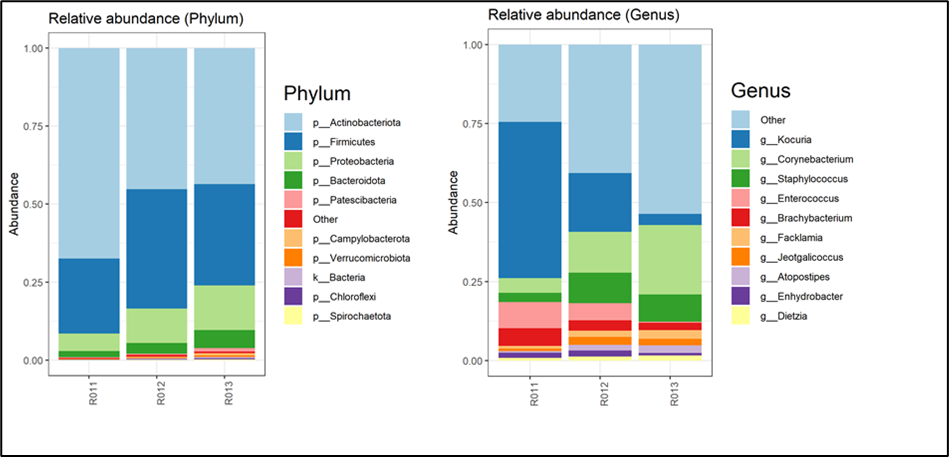


Figure S4. Data from Farm 4. Sample R013 is a milk tank sample containing two milking moments. Please note that the bacterial colony forming units was unusually high in sample R011


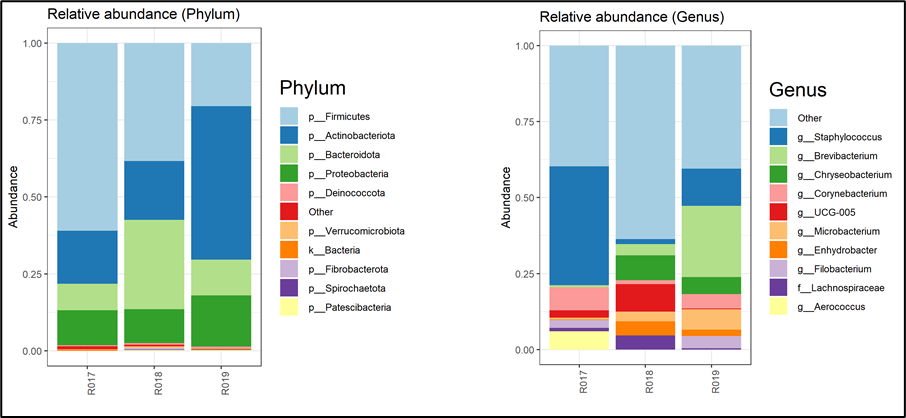


Figure S5. Data from Farm 5. Sample R017 is a milk tank sample containing one milking. Please note that the bacterial colony forming units was unusually high in sample R018. Sample R018 is from a high productive group of goats, while sample R019 is from a group of goats that have been milked for a longer period without a new lambing.


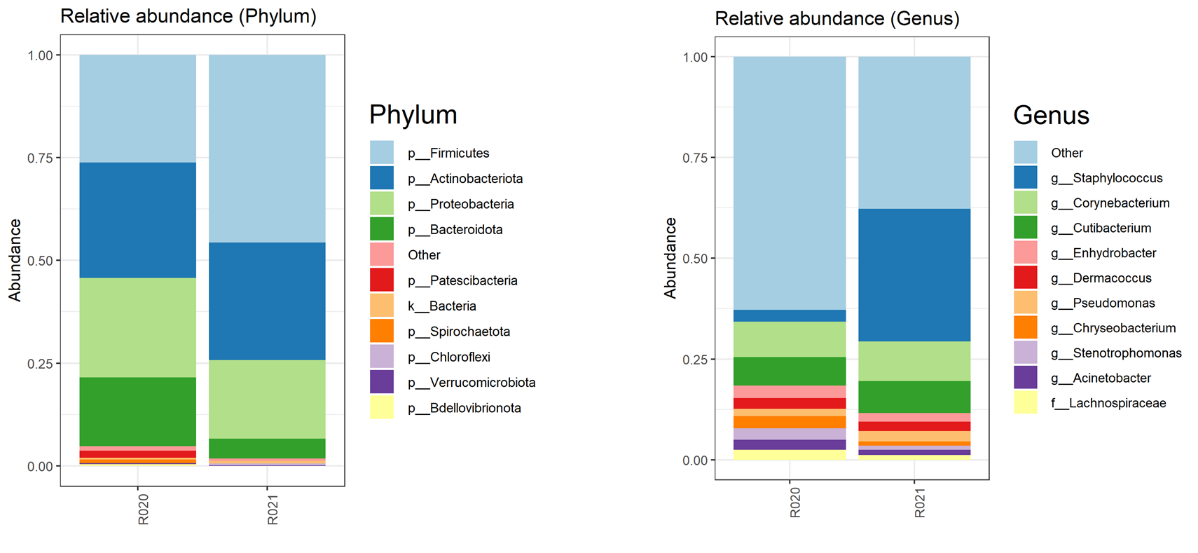


Figure S6. Data from Farm 6.


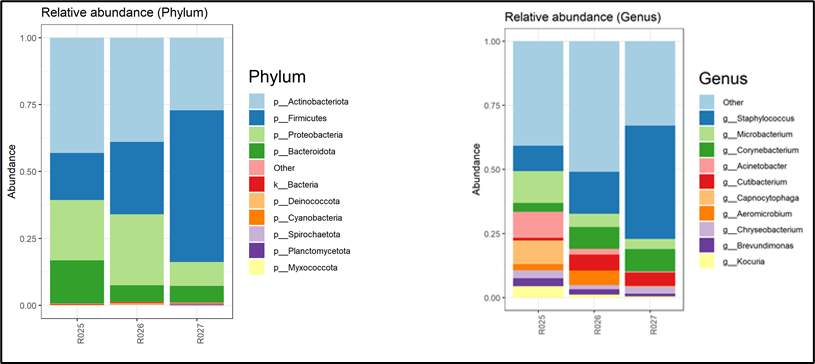


Figure S7. Data from Farm 7. Sample R025 is a milk tank sample containing three milking moments. Sample R026 is from goats lactating on average 1195 days, while sample R027 is from younger higher productive goats about three months lactating.


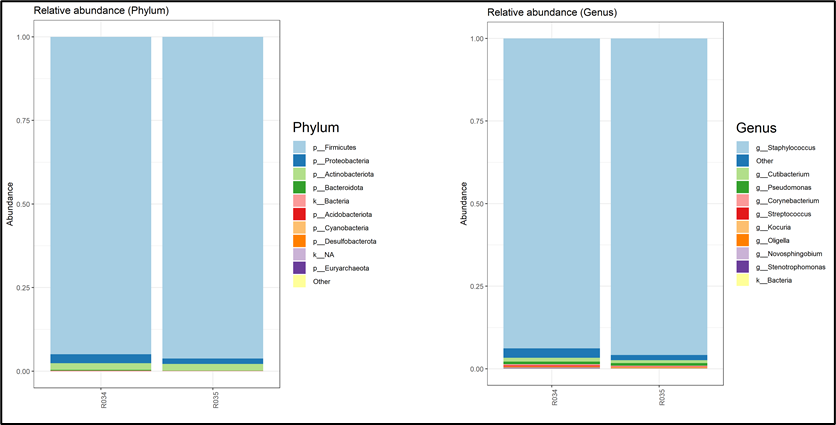


Figure S8. Data from Farm 8. Small farm with seven goats, milked by hand. Please note that the *Staphylococcus* is a human and animal skin bacterium.


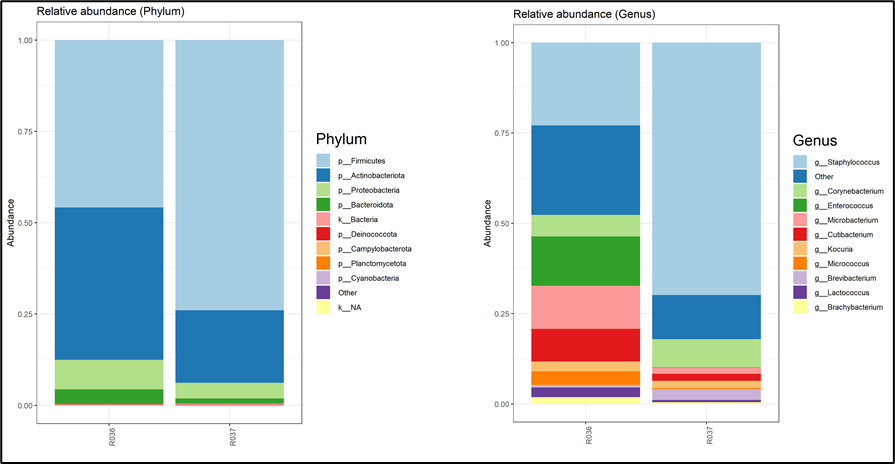


Figure S9. Data from Farm 9.


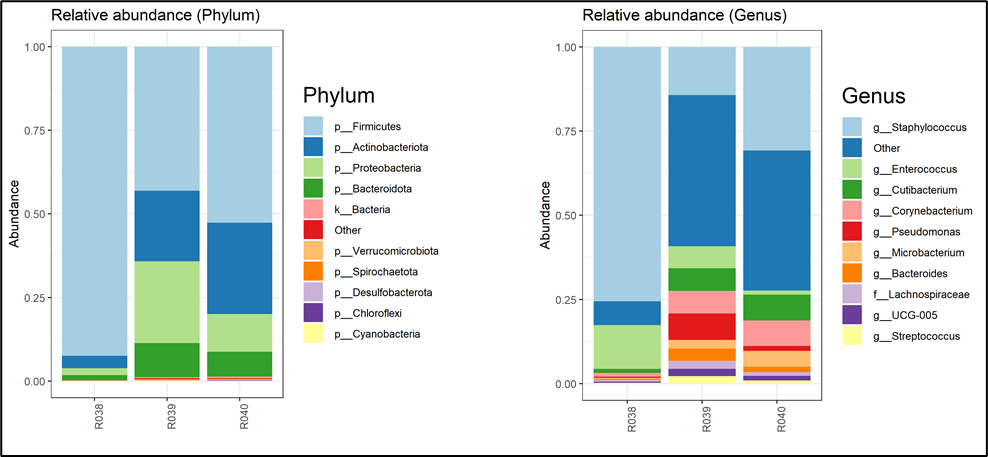


Figure S10. Data from Farm 10. Sample R038 is a milk tank sample.

**Statistical analysis of the diversity of the goat milk microbiome**


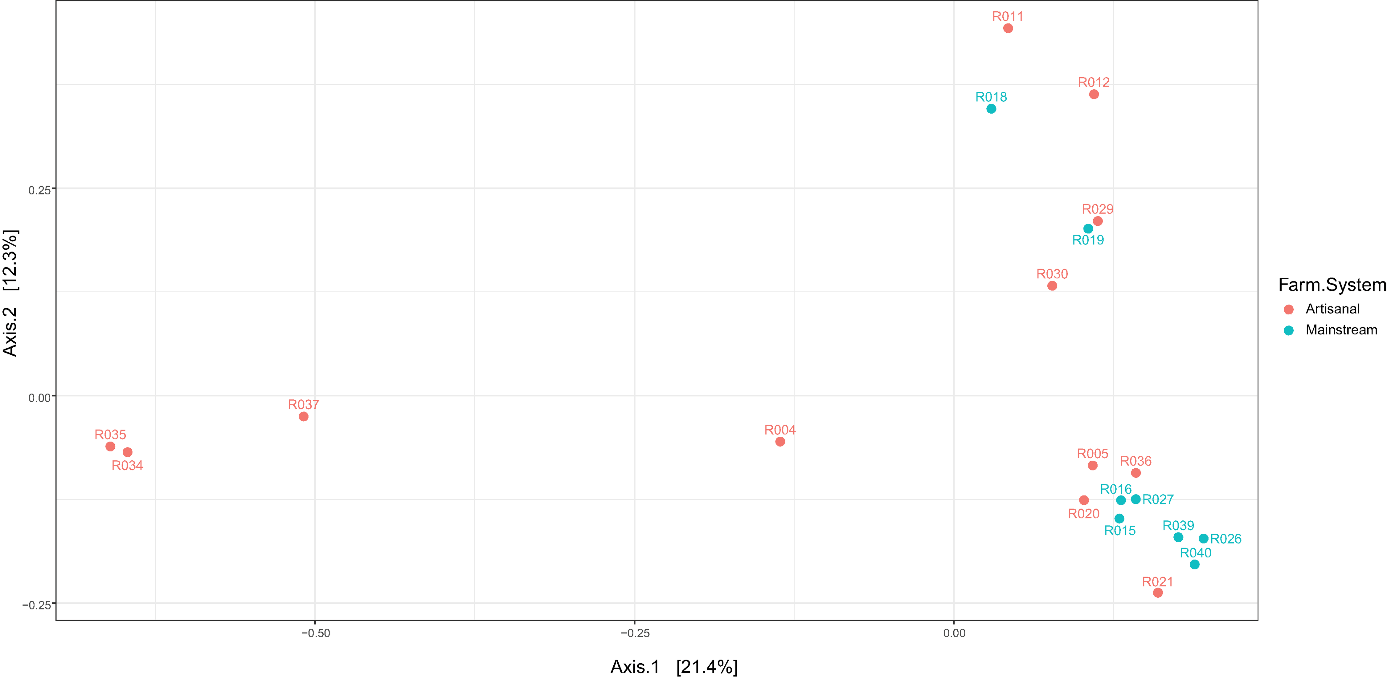


Figure S11. The Principal Coordinates Analysis (PCoA, multidimensional scaling) shows the similarities and dissimilarities of the goat milk microbiota among the farms. The Figure shows that farms of the two management systems do not cluster together, and the variation among the artisanal farms is larger. The Figure shows that 33.6% (21.4% + 12.2%) of the variation is explained by the first two axes. Farm 1: R029, R030; Farm 2: R004, R005; Farm 3: R015, R016; Farm 4: R011, R012; Farm 5: R018, R019; Farm 6: R020, R021; Farm 7: R026, R027; Farm 8: R034, R035; Farm 9: R036, R037; Farm 10: R039, R040.
